# Parent and staff perceptions of racism in a single-center neonatal intensive care unit

**DOI:** 10.1038/s41390-023-02980-w

**Published:** 2024-01-02

**Authors:** Kayla L. Karvonen, Olga Smith, Brittany D. Chambers Butcher, Linda S. Franck, Safyer McKenzie-Sampson, Monica R. McLemore, Matthew S. Pantell, Elizabeth E. Rogers

**Affiliations:** 1 Department of Pediatrics, University of California San Francisco, San Francisco, CA USA; 2 California Preterm Birth Initiative, San Francisco, CA USA; 3 Independent Researcher, Antioch, CA USA; 4 Department of Human Ecology, University of California Davis, Davis, CA USA; 5 Department of Family Health Care Nursing, University of California San Francisco, San Francisco, CA USA; 6 Department of Pediatrics, Stanford University, Palo Alto, CA USA; 7 Department of Child, Family, and Population Health Nursing, University of Washington, Seattle, WA USA

## Abstract

- In alignment with previous literature, NICU parents reported experiencing racism and NICU staff reported witnessing racism in the NICU. Our study also uniquely describes personal experiences with racism by staff in the NICU.
- NICU staff reported witnessing and experiencing racism more often than parents reported.
- Black staff reported witnessing and experiencing more racism than white staff.
- Differences in reporting is likely influenced by variations in lived experience, social identities, psychological safety, and levels of awareness.
- Future studies are necessary to prevent and accurately measure racism in the NICU.

## Introduction

Interpersonal, institutional, and structural forms of racism are core drivers of racial inequities in neonatal outcomes.^1^ Families of infants and staff in Neonatal Intensive Care Units (NICUs) have described experiences with racism,^2,3^ yet detailed data about these experiences are limited. Intersectionality of identity shape experiences of racism and discrimination, yet the extent and variation of experiences with racism by role (i.e., staff or parent), type of staff, lived experience, and other identities is not yet known. We sought to quantify and compare parent and staff perceptions of racism in a single tertiary NICU.

## Methods

The **R**acial and **E**thnic **J**ustice in **O**utcomes in Neonatal **I**ntensive **C**ar**e** (REJOICE) study is a mixed methods study to understand how racism is operationalized in a single-center tertiary care NICU setting by examining differences in standards of care, outcomes, and experiences of individuals of different racial and ethnic identities. Data collected included inpatient parent and staff demographics and their responses to the Everyday Discrimination Scale (EDS) adapted for medical settings (a 7 item, 5 point Likert scale).^4^ The EDS is the most widely used scale measuring racism and discrimination in the clinical context.^4^ The EDS initial question stem was modified to narrow responses to NICU specific encounters. For example, for parents: “Please think about all the care you have received since being admitted to the Neonatal Intensive Care Unit. When getting health care, how often have any of the following things happened to you or your baby because of your race, ethnicity, or color?”. Whereas staff were asked: “Please think about the experiences you have had in the Neonatal Intensive Care Unit. When at work, how often have any of the following things happened to you [or happened to patients] because of your [or their] race, ethnicity, or color?” Each of the 7 items ask about a different form of racism. For example, staff were asked “Do your coworkers (including co-residents, fellows, attendings, nurses, nurse practitioners, respiratory therapists, pharmacists) act as if you are not smart?” Available response options to all questions were “never”, “rarely”, “sometimes”, “most of the time”, or always”. Multiple strategies were used to recruit participants, including in-person outreach by a racially diverse research team, remote outreach by stand-alone flyers and signage in the unit, and virtual outreach by email. Data were collected in 2021–2022 via electronic survey available in English and Spanish. Participants completed the survey online at any time during hospitalization or employment within the study period. Descriptive statistics and Kruskal–Wallis tests were used to compare parent’s own experiences with racism, witnessed parent experiences with racism reported by staff, and staff’s own experiences with racism. Chi-squared testing was used to compare demographics between staff or parent status. Unadjusted ordered logistic regressions were fit to test the association of racial/ethnic groups and total EDS score, compared to the white parent or staff group. Methods and protocols for the study were approved by the Institutional Review Board of the University of California, San Francisco.

## Results

**Fig. 1:**
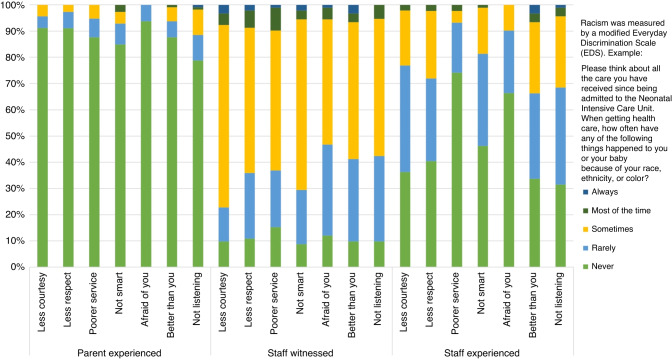
Frequency of parent and staff experiences with racism.

113 parents and 92 staff completed the survey. Most parents identified as female (77.9%), Hispanic/Latinx, white, and Asian (46.4%, 25.0%, and 14.3%). Black parents and staff were in the minority (6.3% and 13.0%). Most staff identified as female (84.8%), as white and Asian (46.7%, 25.0%), and as nurses and medical trainees (50.0%, 16.3%, Table 1). Participant demographics mirrored the wider NICU demographics. Most parents reported never or rarely experiencing racism in the NICU (all median item scores 1). Staff more often witnessed parents experiencing racism than parents reported experiencing racism (median item scores 2–3, median total score [MTS] 19 [interquartile range [IQR] 15–21] vs. MTS 7 [IQR 7–8] *p* < 0.001). Staff rarely to sometimes experienced racism, which was more frequent than parents reported (median item scores 1–3, MTS 12 [IQR 8–15] vs. MTS 7 [IQR 7–8] *p* < 0.001, Fig. 1). There were no differences in MTS by age or language among staff or parents. Staff-witnessed racism varied by role with highest levels reported by trainees (*p* = 0.03). Staff experiences of discrimination measured by MTS varied by sex, with highest levels reported by males (*p* = 0.04). Parent experiences did not vary by race/ethnicity or sex. Black staff witnessed and experienced more racism their white colleagues (OR 5.2 95% CI 1.6–17.1 *p* < 0.01, OR 10.8 95% CI 3.0–38.6 *p* < 0.001). Asian and Hispanic/Latinx staff also reported experiencing more racism compared to white staff (OR 3.4 95% CI 1.4–8.2 *p* < 0.01, OR 5.2 95% CI 1.1–23.7 *p* = 0.04, Table 2).

**Table 1:** Participant characteristics.

		Parent	Staff
		*n* (%)	*n* (%)
Age (years)^a^			
	<24	20 (17.0)	0 (0)
	25–34	42 (37.2)	39 (42.4)
	35–44	48 (42.5)	26 (28.3)
	45+	3 (2.7)	27 (29.4)
Sex			
	Female	88 (77.9)	78 (84.8)
	Male	25 (22.1)	14 (15.2)
Language^a^			
	English	46 (40.7)	92 (100)
	Other	67 (59.3)	0 (0)
Race/ethnicity^a^			
	Hispanic/Latinx	52 (46.4)	5 (5.4)
	Black	7 (6.3)	12 (13.0)
	White	28 (25.0)	43 (46.7)
	Asian	16 (14.3)	23 (25.0)
	American Indian/Alaska Native	0 (0)	1 (1.1)
	Native Hawaiian/ Pacific Islander	1 (.9)	6 (6.5)
	Multiracial (>1 race)	8 (7.1)	2 (2.2)
Role			
	Nurse		46 (50.0)
	Trainee (resident or fellow)		15 (16.3)
	Nurse Practitioner or Hospitalist		10 (10.9)
	Attending		9 (9.8)
	Respiratory Therapist		8 (8.7)
	Other Staff		4 (4.4)
Total		113 (100)	92 (100)

^a^*p*-value < 0.05.

**Table 2:** Odds of racism reported by parents and staff by race/ethnicity^a,b^.

	Parent experienced		Staff witnessed		Staff experienced	
Race/ethnicity	OR	(95% CI)	OR	(95% CI)	OR	(95% CI)
Hispanic/Latinx	1	(0.3–2.8)	0.6	(0.1–2.5)	**5.2**	**(1.1**–**23.7)**
Black	3.4	(0.6–18.6)	**5.2**	**(1.6**–**17.1)**	**10.8**	**(3.0**–**38.6)**
Asian	1.6	(0.4–6.0)	1.1	(0.5–2.7)	**3.4**	**(1.4**–**8.2)**
Multiracial	3.1	(0.7–13.8)	^b^	^b^	^b^	^b^
White	reference	reference	reference	reference	reference	reference

^a^OR bolded if *p* < 0.05.

^b^Race/ethnicity excluded if *n* < 5.

## Discussion

In this study, NICU parents and staff report witnessing and experiencing racism and discrimination in the NICU, in alignment with previous literature.^2,3,5^ Families and staff have previously reported disparate neglectful, judgmental, and systemic barriers to care in the NICU.^2^ In other healthcare settings, physicians of color have highlighted a variety of experiences with racism that have impacted their mental health and sense of well-being and interfered with professional advancement and professional quality of life.^6^ Our study uniquely found that significant differences exist between parent and staff perceptions of racism; NICU staff reported witnessing and experiencing racism more often than parents of infants in the NICU.

We suspect etiologies of reported differences in witnessing and experiencing racism by parents and staff are multifactorial. Parents were surveyed during hospitalizations and thus, they may be reluctant to report racism due to insufficient psychological safety in the setting of extreme power dynamics. Alternatively, parents may be unaware of differences in care if they have limited opportunities to observe care of other families due to language or physical barriers i.e., single hospital rooms. Training in diversity, equity, and inclusion (DEI) and antiracism is increasing in the medical field and thus staff may be more sensitive to report racism. Additionally, in contrast to local studies that describe disrespectful and stressful healthcare experiences by women of color, we found no statistical differences in parent experience of racism by racial/ethnic group.^7^ There were very few Black parents in this study, and we suspect we may have had inadequate power to quantitatively detect anti-Black racism. In regards to staff, however, Black staff were more likely to witness and experience racism compared to white staff, in alignment with detailed accounts of racism in other medical settings.^6^ All racialized groups were more likely to report experiencing racism compared to white staff, an unsurprising finding given a legacy of white supremacist ideology in the U.S. Black staff were the only group more likely to report witnessing racism compared to white staff, which may reflect a different threshold for detecting and reporting racism in part due to lived experience and/or higher levels of anti-Black racism in the NICU.

Social identities are diverse, intersectional, and inform experiences of discrimination.^8^ We also found heterogeneity in staff perceptions of discrimination by role and sex. Trainees were more likely than staff in other roles to report witnessing, but not experiencing racism. By nature of the role, trainees tend to be younger with less experience in hospital environments and thus, bring a fresh perspective on hierarchal systems and they may be more comfortable speaking out against them. Trainees may also experience differential treatment through the medical hierarchy and be more sensitive to reporting differential treatment. Male staff were also more likely to report discrimination. We suspect intersectionality is relevant here as well, as male staff participants were mostly trainees and identified as a racially minoritized group. Notably our staff sample was primarily female, with males only making up 15% of our sample, a trend similar to the general field of pediatrics.

Limitations of the study include a lack of detail on other identities that may inform perspectives and a generally small sample size with limited power to detect differences between those with different identities as evidenced by wide confidence intervals when comparing across racial/ethnic groups indicating imprecision of effect estimates. Future studies should investigate nativity, immigration status, and more rich detail regarding racial and ethnic group and gender identities.^9^ Geographic location and socioeconomic status may also inform experiences, but were not collected in this study.^10,11^ Other studies have surmised that variable anti-bias education, levels of awareness, and personal insight may also be significant modifiers to consider as it relates to parent and staff reports of discrimination.^5^ Although EDS is a commonly used measurement of racism and discrimination, more sophisticated quantitative measures of racism have been developed and validated in other clinical settings.^12^ Although adapted versions of EDS has been used and validated across racial/ethnic groups, this version was validated in a sample of African-American patients, a small proportion of our sample.

Future studies to better understand the experience of racism for parents of infants in NICUs and in their home communities are needed.^13^ Vicarious forms of racism experienced by staff of color also have impact on their mental health and well-being and should additionally be explored and addressed in this setting.^6^ Specifically, qualitative studies investigating staff and parent perspectives are necessary to provide more detailed underlying themes and mechanisms driving differences in perceptions of racism. This study will inform additional considerations when measuring and addressing racism in the NICU to improve family-centered care and work environments. Although measuring and addressing racism is an important proximal goal, we recognize that the ultimate goal should be to reform societal and institutional cultures to prevent harms inflicted secondary to racism before they occur.

## Conclusion

Overall, parents and staff experienced and witnessed racism in the NICU. Staff reported witnessing and experiencing more racism and discrimination compared to parents in the NICU, who reported infrequently experiencing racism and discrimination. Differences in reporting is likely influenced by variations in lived experience, identities, psychological safety, and levels of awareness. Future studies are necessary to prevent and accurately measure racism in the NICU.

## Data Availability

The datasets generated during the current study are available from the corresponding author on reasonable request.
